# Accurate method for measuring arterial pulse wave velocity by cardiovascular magnetic resonance

**DOI:** 10.1186/1532-429X-14-S1-O12

**Published:** 2012-02-01

**Authors:** El-Sayed Ibrahim

**Affiliations:** 1 Department of Radiology, University of Florida, Jacksonville, FL, USA

## Summary

A modified version of the flow-area (QA) method for measuring pulse wave velocity (PWV) from CMR images is introduced. The new ‘flow-time-area’ (QTA) method is more robust to errors in area measurements than the QA method, which is mathematically proved and demonstrated in pulmonary hypertension study. PWV was correlated to measurements from catheterization, echo, and CMR. The effect of image resolution, inter-, and intra-observer variabilities were studied. The results showed superiority of the QTA method over QA method.

## Background

The flow-area (QA) method has potential importance for measuring pulse wave velocity (PWV) from CMR images, especially in the pulmonary artery (PA). Nevertheless, the QA method is highly affected by errors in area measurements. In this work, we introduce the ‘flow-time-area’(QTA) method, a modified version of QA, which has benign behavior to area measurements inaccuracies.

## Methods

In QTA, area and flow changes are calculated separately w.r.t. time, from which PWV is calculated by dividing the two slopes. The following sections show that QTA is more accurate than than QA.

### 1.Mathematical proof

In QA, flow measurements, q, are related to area measurements, a, by linear least-squares fit:q=ua+v, where PWV_QA_ = u. By solving the normal equation⇒ PWV_QA_=(m∑a_i_q_i_-∑a_i_∑q_i_)/(m∑(a_i_)^2^-(∑a_i_)^2^), where m= #measurements. In QTA, two lines are formed: q=u_1_t+v_1_ and a=u_2_t+v_2_, from which⇒ PWV_QTA_=(m∑t_i_q_i_-∑t_i_∑q_i_)/(m∑t_i_a_i_-∑t_i_∑a_i_). Under perfect linear relationship between q and a, PWV_QA_=PWV_QTA_=u. We analyze the case of positive error in area measurement (Δa) at point j. j is assumed >m/2, which reduces PWV and the accuracy measure M=PWV/ΔPWV-1, where ΔPWV is the difference between exact and measured PWV. For zero measurement error, M→∞. By mathematical manipulation, M_QA_=(N_1_+N_2_)/(D_1_+D_2_) and M_QTA_=N_1_/D_1_, where N_1_=u_2_(m∑(t_i_)^2^-(∑t_i_)^2^), N_2_=D_1_=(mt_j_-∑t_i_)Δa, and D_2_=(m-1)(Δa)^2^/u_2_. It can be shown that N_1_D_2_-N_2_D_1_=(t_i_-t_k_)^2^≥0 (summation over i>k; i,k≠j), which proves that M_QA_≤M_QTA_.

### 2.Application

**Figure 1:**
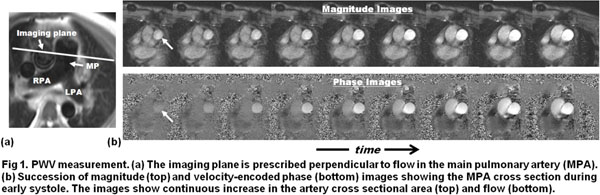


50 human subjects (25 PA hypertension (PAH) and 25 volunteers) were scanned on Siemens 3T scanner. Two sets of velocity-encoded images were acquired with resolutions 0.7mm and 1.4mm (Fig. 1). PA boundary was semi-automatically determined by two observers. PWV was calculated by QTA and QA. The measurements were compared using t-test. Normalized least-squares errors were calculated. Correlation analysis was conducted between PWV and mean PA pressure (mPAP), pulmonary vascular-resistance (PVR), and cardiac-index (CI). Bland-Altman analysis was conducted to measure inter- and intra-observer variabilities.

**Figure 2:**
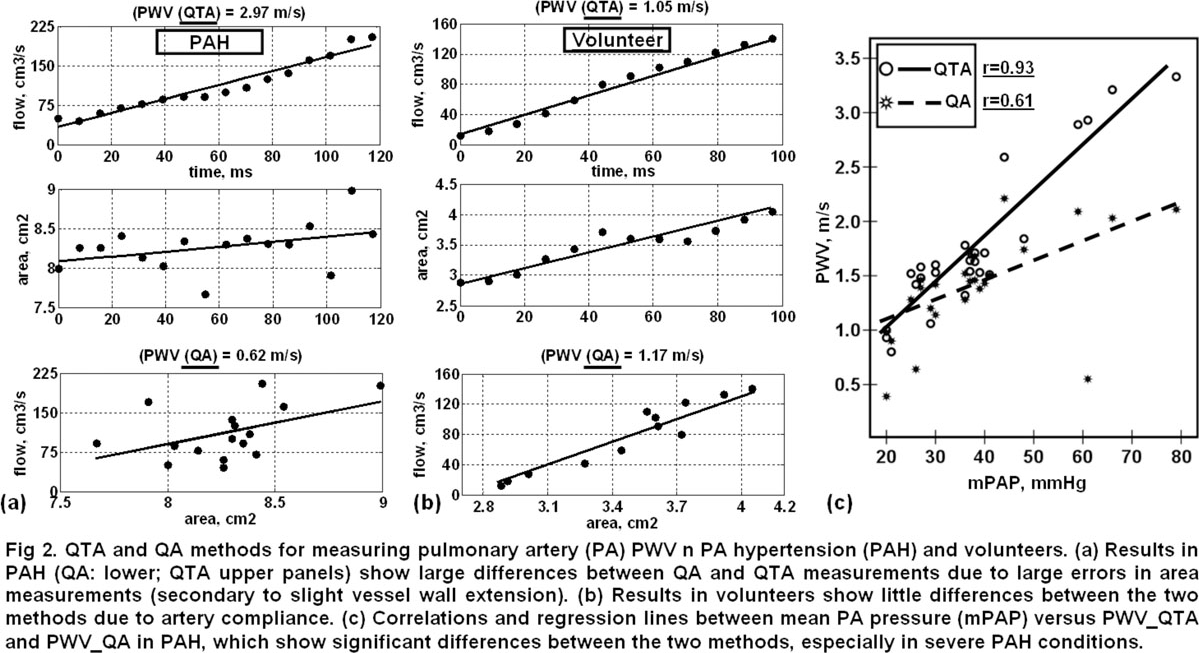


## Results

PWV_QTA_ and PWV_QA_ showed significant differences in PAH(p<.005), and less significant in volunteers (p=.01). The following results are for PAH: Normalized fitting-errors were 0.16/0.31for QTA/QA. Correlation coefficients between PWV versus mPAP, PVR, and CI were 0.93/0.61, 0.86/0.65, -0.71/-0.62 for QTA/QA. Standard deviations of differences between high- and low-resolution measurements were 0.19/0.41 m/s for QTA/QA. Correlation coefficients between low-resolution PWV and mPAP were 0.87/0.4 for QTA/QA. Bland-Altman showed minimal inter- and intra-observer variabilities.

## Conclusions

The QTA method is more accurate than QA, because area measurement errors are not propagated to flow. The improved QTA accuracy is imperative when changes in artery cross-sectional area are minimal(e.g. PAH), when spatial resolution is low, or automatic vessel segmentation is implemented. Thus, QTA should be adopted for accurate PWV estimates.

## Funding

James & Esther King Grant # 09KN-03-23138.

